# Genome Analysis of *Thermosulfurimonas dismutans*, the First Thermophilic Sulfur-Disproportionating Bacterium of the Phylum *Thermodesulfobacteria*

**DOI:** 10.3389/fmicb.2016.00950

**Published:** 2016-06-17

**Authors:** Andrey V. Mardanov, Alexey V. Beletsky, Vitaly V. Kadnikov, Alexander I. Slobodkin, Nikolai V. Ravin

**Affiliations:** ^1^Institute of Bioengineering, Research Center of Biotechnology of the Russian Academy of SciencesMoscow, Russia; ^2^Winogradsky Institute of Microbiology, Research Center of Biotechnology of the Russian Academy of SciencesMoscow, Russia

**Keywords:** sulfur disproportionation, thermophile, *Thermodesulfobacteria*, thiosulfate, genome sequence

## Abstract

*Thermosulfurimonas dismutans* S95^T^, isolated from a deep-sea hydrothermal vent is the first bacterium of the phylum *Thermodesulfobacteria* reported to grow by the disproportionation of elemental sulfur, sulfite, or thiosulfate with carbon dioxide as the sole carbon source. In contrast to its phylogenetically close relatives, which are dissimilatory sulfate-reducers, *T. dismutans* is unable to grow by sulfate respiration. The features of this organism and its 2,1 Mb draft genome sequence are described in this report. Genome analysis revealed that the *T. dismutans* genome contains the set of genes for dissimilatory sulfate reduction including ATP sulfurylase, the AprA and B subunits of adenosine-5′-phosphosulfate reductase, and dissimilatory sulfite reductase. The oxidation of elemental sulfur to sulfite could be enabled by APS reductase-associated electron transfer complex QmoABC and heterodisulfide reductase. The genome also contains several membrane-linked molybdopterin oxidoreductases that are thought to be involved in sulfur metabolism as subunits of thiosulfate, polysulfide, or tetrathionate reductases. Nitrate could be used as an electron acceptor and reduced to ammonium, as indicated by the presence of periplasmic nitrate and nitrite reductases. Autotrophic carbon fixation is enabled by the Wood–Ljungdahl pathway, and the complete set of genes that is required for nitrogen fixation is also present in *T. dismutans*. Overall, our results provide genomic insights into energy and carbon metabolism of chemolithoautotrophic sulfur-disproportionating bacterium that could be important primary producer in microbial communities of deep-sea hydrothermal vents.

## Introduction

The biogeochemical sulfur cycle in the modern biosphere depends on activities of different anaerobic and aerobic microorganisms. One particular group of sulfur-metabolizing prokaryotes, i.e., the bacteria that disproportionate sulfur compounds, simultaneously perform the sulfur oxidation and reduction (Bak and Cypionka, 1987; Bak and Pfennig, 1987; Thamdrup et al., 1993). In this process, elemental sulfur, thiosulfate or sulfite each serves as both an electron donor and acceptor and became converted into sulfate and hydrogen sulfide:

$$4\;S^{0}+4H_{2}O\to SO_{4}^{2-}+3HS^{-}+5H^{+}$$

$$\Delta G\circ \prime =+10.3\,\;kJ\,\;mol^{-1}\,\;S^{0}\,$$

$$S_{2}O_{3}^{2-}+H_{2}O\to SO_{4}^{2-}+HS^{-}+H^{+}$$

$$\Delta G\circ \prime =-22.3\,\;kJ\,\;mol^{-1}\,\;S_{2}O_{3}^{2-}$$

$$4SO_{3}^{2-}+H^{+}-3SO_{4}^{2-}+HS^{-}$$

$$\Delta G\circ \prime =-58.9kJ\,\;mol^{-1}\,\;SO_{3}^{2-}$$

The disproportionation of elemental sulfur is endergonic under standard conditions and proceeds only under low concentrations of hydrogen sulfide, which is achieved in the environment by the precipitation of sulfide with iron or by rapid oxidation. Disproportionation of inorganic sulfur compounds is of environmental significance in marine sediments (Jørgensen, 1990), and could be one of the earliest microbial processes dating back to 3.4 Ga (Philippot et al., 2007).

The process of disproportionation of inorganic sulfur compounds was described for about twenty species of the class *Deltaproteobacteria*, most of which are dissimilatory sulfate reducers (Finster, 2008). Among them, there are two thermophilic species, *Dissulfuribacter thermophilus* and *Dissulfurimicrobium hydrothermale* (Slobodkin et al., 2013, 2016). Outside *Deltaproteobacteria*, the ability to disproportionate sulfur compounds has been shown for three species of the phylum *Firmicutes* (genera *Desulfotomaculum* and *Dethiobacter*) and for the gamma-proteobacterium *Pantoea agglomerans* (Jackson and McInerney, 2000; Obraztsova et al., 2002; Nazina et al., 2005). Recently, the capacity for sulfur disproportionation has been reported for members of the phylum *Thermodesulfobacteria* – *Thermosulfurimonas dismutans* (Slobodkin et al., 2012) and *Caldimicrobium thiodismutans* (Kojima et al., 2016).

The metabolic pathways enabling disproportionation of thiosulfate and sulfite have been partly resolved in biochemical studies (Kramer and Cypionka, 1989; Frederiksen and Finster, 2003, 2004; Finster, 2008), but the enzymatic machinery of elemental sulfur disproportionation remains unclear. Complete genome sequences of several sulfur-disproportionating microorganisms are publically available; however, the analysis of genomic data in relation to mechanisms underlying the disproportionation of sulfur compounds has so far only been made for *Desulfocapsa sulfoexigens* (Finster et al., 2013).

Here, we present the results of sequencing and analysis of *Thermosulfurimonas dismutans* S95^T^ genome that provided insights into the mechanisms of disproportionation of sulfur compounds. *T. dismutans*, isolated from deep-sea hydrothermal vent, is an anaerobic thermophilic bacterium which is able to grow chemolithoautotrophically by disproportionation of elemental sulfur, thiosulfate, and sulfite (Slobodkin et al., 2012). Unlike the majority of deltaproteobacterial sulfur disproportionators, *T. dismutans* is unable to respire sulfate. Elemental sulfur is abundant in some marine hydrothermal vents (Nakagawa et al., 2006), and its disproportionation by thermophilic bacteria could be an important process of primary production of organic matter in these ecosystems.

## Materials and Methods

### Cultivation of *T. dismutans*

*Thermosulfurimonas dismutans* strain S95^T^ was isolated from a sample of the actively venting hydrothermal sulfidic chimney-like deposit located at the Mariner hydrothermal field (1910 m depth) on the Eastern Lau Spreading Center, Pacific Ocean and was maintained in the culture collection of the Laboratory of Hyperthermophilic Microbial Communities, Winogradsky Institute of Microbiology, Russian Academy of Sciences (Slobodkin et al., 2012). To obtain biomass for genome sequencing, the strain was grown in sealed bottles as previously described (Slobodkin et al., 2012) in anaerobic, bicarbonate-buffered marine liquid medium with 101 kPa of H_2_:CO_2_ (80%:20%) in the headspace and 10 mM Na_2_S_2_O_3_ as the electron acceptor. The medium composition and preparation techniques were described previously (Slobodkin et al., 2012). The pH of the medium was 6.5–6.8 and the incubation temperature was 65°C. Cells were collected by centrifugation and then genomic DNA was isolated by SDS-CTAB method (Milligan, 1998). In order to determine if direct cell contact with sulfur is necessary for growth, *T. dismutans* was grown in the medium above except that thiosulfate was replaced with elemental sulfur entrapped into alginate beads. The technique for forming the sulfur alginate beads is as described previously (Gavrilov et al., 2012), except that a 1% (w/v) suspension of elemental sulfur (sublimed, Sigma) was used in place of ferrihydrite.

### Genome Sequencing and Annotation

The *T. dismutans* S95^T^ genome was sequenced with a Roche Genome Sequencer (GS FLX), using the Titanium XL+ protocol for a shotgun genome library. The GS FLX run resulted in the generation of about 143 Mb of sequences with an average read length of 635 bp. The GS FLX reads were *de novo* assembled using Newbler Assembler version 2.9 (454 Life Sciences, Branford, CT, USA). The draft genome of *T. dismutans* S95^T^ consists of 61 contigs longer than 500 bp, with a total contig length of 2,119,932 bp.

Gene calling, annotation and analysis were performed for all contigs longer than 500 bp. Coding genes were annotated using the RAST server (Brettin et al., 2015). The annotation was manually corrected by searching the National Center for Biotechnology Information (NCBI) databases. The tRNAscan-SE tool (Lowe and Eddy, 1997) was used to find and annotate tRNA genes, whereas ribosomal RNA genes were found by RNAMMER server (Lagesen et al., 2007). Signal peptides were predicted using Signal P v.4.1 for Gram-negative bacteria^1^. The N-terminal twin-arginine translocation (Tat) signal peptides were predicted using PRED-TAT^2^, the transmembrane helices – with TMHMM Server v. 2.0^3^.

For phylogenetic analysis of the catalytic A subunits of molybdopterin oxidoreductases the *T. dismutans* proteins TDIS_0362, TDIS_0614, TDIS_0652, TDIS_1010, and TDIS_1816 were used along with consensus sequences of the A subunits of tetrathionate reductases (Ttr), formate dehydrogenases (Fdh), thiosulfate or polysulfide reductases (Psr), and DMSO reductases (Dmsr), defined in Yanyushin et al. (2005). Amino acid sequences were aligned using MUSCLE (Edgar, 2004). Ambiguously aligned sites were removed using trimAl (Capella-Gutiérrez et al., 2009) before the phylogenetic reconstruction. The maximum likelihood phylogenetic tree was computed by PhyML 3.1 (Guindon et al., 2010), using the gamma model of rate heterogeneity (four discrete rate categories, an estimated alpha-parameter) and LG substitution matrix. The support values for the internal nodes were estimated by the approximate Bayesian method.

### Nucleotide Sequence Accession Number

The annotated genome sequence of *T. dismutans* has been deposited in the GenBank database under accession no LWLG00000000.

## Results

### General Features of the Genome

Sequencing and assembly of *T. dismutans* draft genome resulted in 61 contigs longer than 500 bp, with a total contig length of 2,119,932 bp. The G+C content of the genome is 50.1%. A single 16S-23S-5S rRNA operon and 48 tRNA genes coding for all of the 20 amino acids were identified.

Using a combination of coding potential prediction and similarity searches, 2159 protein-coding genes were predicted. Of these, 1458 genes were functionally assigned with different degrees of generalization and confidence, while the function of the remaining 701 genes could not be predicted from the deduced amino acid sequences. The properties and the statistics of the genome are summarized in **Table 1**. Consistent with its affiliation to the phylum *Thermodesulfobacteria*, *T. dismutans* shares more than half of the proteome with that of its closest relative with sequenced genome, *Thermodesulfatator indicus* (1326 proteins).

**Table 1:** General features of the genome.

Attribute	Value
Size (bp)	2,119,932
G+C content (%)	50.12
Coding region (%)	93.03
Total genes	2210
rRNA genes	3
tRNA genes	48
Protein-coding genes, of them:	2159
Genes with predicted functions	1458
Genes assigned to COGs	1443
Genes with signal peptides	86
Genes specific to *T. dismutans*	260

### Metabolism of Inorganic Sulfur Compounds

Although *T. dismutans* cannot grow by sulfate reduction, its genome contains the complete set of genes for dissimilatory sulfate reduction (Bradley et al., 2011; Pereira et al., 2011), including sulfate adenylyltransferase (TDIS_1516), manganese-dependent inorganic pyrophosphatase (TDIS_1154), APS reductase subunits AprA and AprB (TDIS_1513 and TDIS_1514), the subunits of dissimilatory sulfite reductase DsrABD (TDIS_1700, TDIS_1701, TDIS_1702), and distantly encoded DsrC (TDIS_1619). All of these predicted proteins lack signal peptides and transmembrane helices and were predicted to be located in the cytoplasm. The sulfate-reduction pathway could be linked to the membrane by sulfite reductase-associated electron transfer complex DsrMKJOP (TDIS_0546- TDIS_0542). Two of its subunits, DsrM and DsrP were predicted to contain transmembrane domains, while the iron-sulfur protein DsrO contains a Tat signal peptide enabling its translocation across the periplasmic membrane. A three-gene operon (TDIS_1512- TDIS_1510) encoding the subunits QmoA, QmoB, and QmoC of APS reductase-associated electron transfer complex QmoABC is located immediately downstream of the *aprBA* operon. The QmoA subunit contains a conserved FAD-binding site and the four cysteine cluster that binds an Fe–S center. QmoB contain FAD-binding site, 4Fe–4S double cluster binding domain and C-terminal domain similar to the delta subunit of methyl-viologen-reducing hydrogenase. The QmoC contains 4Fe–4S dicluster and transmembrane domain, thus linking the QmoABC complex to the cytoplasmic membrane. In sulfate reducing bacteria, QmoABC transfers electrons from the quinone pool to AprAB (Pires et al., 2003; Venceslau et al., 2010). Here, the electron transport could proceed in the opposite direction. This complex could also play a role in the oxidation of sulfur compounds to sulfite, as discussed below. The *T. dismutans* genome also encodes rodanese-like sulfurtransferase (TDIS_0247) that could participate in the thiosulfate and/or sulfur disproportionation, although the actual physiological role of this enzyme is unclear. Sulfur transport could be facilitated by sulfotransferase TDIS_0343, sulfur relay protein TusA (TDIS_0895) and integral membrane protein TDIS_0896.

Our additional physiological experiments revealed that *T. dismutans* is capable of sustained growth (at least four subsequent 5% v/v transfers) via disproportionation of elemental sulfur entrapped in alginate beads (a nominal molecular mass cutoff of 12 kDa). This finding indicates that the direct contact of the cells to solid elemental sulfur is not required for growth, and the actual substrate for disproportionation is not the poorly soluble elemental sulfur, but most likely soluble polysulfides abiotically formed under these conditions.

**FIGURE 1:**
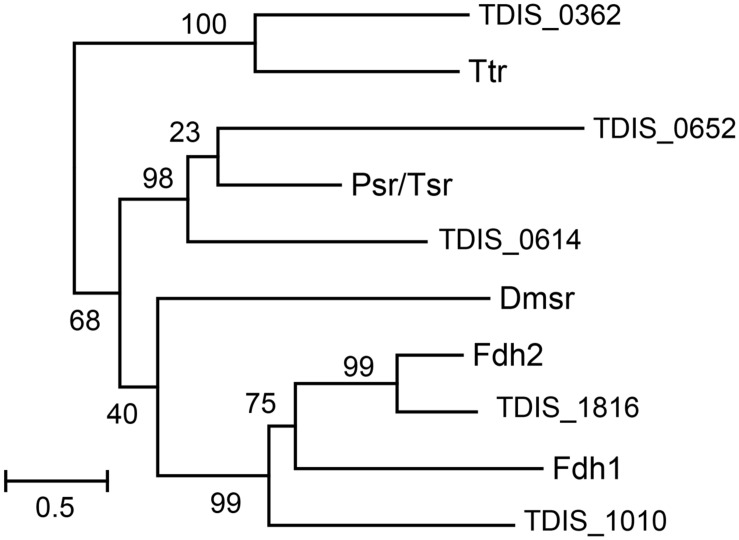
**Phylogenetic tree of the catalytic A subunits of molybdopterin oxidoreductases**. Abbreviations: Ttr, tetrathionate reductases; Psr/Tsr, polysulfide and thiosulfate reductases; Dmsr, DMSO reductase; Fdh1 and Fdh2, formate dehydrogenases. Numbers at nodes represent the support values estimated by an approximate Bayesian method. The scale bar represents the average number of substitutions per site.

Analysis of the *T. dismutans* genome revealed several membrane-linked oxidoreductases that could be involved in reduction of inorganic sulfur compounds. Among them there are four putative molybdopterin oxidoreducases of the Psr/Psh family. Such complexes typically consist of a molybdopterin-binding catalytic A subunit, an electron-transfer B subunit with an [Fe–S] cluster, and a membrane-anchor C subunit. Phylogenetic analysis of their catalytic A subunits (**Figure 1**) allowed to assign them the functions of tetrathionate reductase (TDIS_0362), formate dehydrogenase (TDIS_1010 and TDIS_1816), and thiosulfate or polysulfide reductases (TDIS_0614 and TDIS_0652). All of these catalytic subunits, except for formate dehydrogenase TDIS_1010, contain N-terminal twin-arginine translocation (Tat) signal peptides, indicating that these oxidoreductases operate on the periplasmic side of the membrane. The presence of hypothetical thiosulfate reductase, capable of producing sulfide and sulfite from thiosulfate, could explain the ability of *T. dismutans* to grow by disproportionation of thiosulfate.

The reduction of tetrathionate could be also enabled by the function of octaheme *c*-type cytochrome tetrathionate reductase (TDIS_1882). This is presumably a periplasmic protein linked to the cytoplasmic membrane by nearby encoded cytochrome *b* subunit (TDIS_1883) containing multiple transmembrane helices. *In vitro* studies of octaheme tetrathionate reductase from *Shewanella oneidensis* suggested a multifunctional role for these enzymes able to catalyze the reduction of tetrathionate, nitrite and hydroxylamine (Atkinson et al., 2007). The Sox system operating in aerobic sulfur oxidizing bacteria is missing in *T. dismutans*.

### Alternative Electron Donors and Acceptors

Analysis of the *T. dismutans* genome revealed additional potential metabolic capabilities of this bacterium. The ability to use nitrate as an electron acceptor was suggested by the presence of an operon *napMADGH* (TDIS_0603- TDIS_0599) encoding periplasmic nitrate reductase. This complex includes the catalytic large subunit NapA, small tetraheme cytochrome *c* subunit NapM, electron transfer subunit NapG with 4Fe–4S double cluster binding domain, membrane anchor NapH and cytoplasmic chaperone NapD. The gene order is similar to that in the *napCMADGH* operon in sulfate and nitrate-reducing deltaproteobacterium *Desulfovibrio desulfuricans* (Marietou et al., 2005), although the small cytochrome NapC was not identified in *T. dismutans*. The nitrate reductase seems to localize in the periplasmic space, as indicated by the presence of N-terminal targeting sequences in NapM, NapA, and NapG subunits. Nitrite, produced from nitrate by this reductase could be further reduced to ammonium by dissimilatory nitrite reductase. This enzyme complex includes the multiheme membrane-bound cytochrome *c* subunit NrfH (TDIS_1141), the membrane subunit NrfD (TDIS_1142), the 4Fe–4S ferredoxin subunit NrfC (TDIS_1143), and the cytochrome c family protein with three heme motifs (TDIS_1144). The presence of N-terminal twin-arginine translocation (Tat) signal peptide in NrfC suggests the periplasmic location of nitrite reductase. We did not identify an apparent homolog of the catalytic NrfA subunit, but since the *nrf* operon is located at the end of a contig, this gene may be split and not found in the assembly. Alternatively, catalytic function could be performed by one of periplasmic multiheme cytochromes (e.g., TDIS_0311, TDIS_1112, and TDIS_1483) or the above-mentioned octaheme tetrathionate reductase.

The ability to reduce nitrate or nitrite was not reported in the original description of *T. dismutans* (Slobodkin et al., 2012), but the genome data prompted to reevaluate this trait. Indeed, our experiments have shown that *T. dismutans* is capable of growing with elemental sulfur as an electron donor and nitrate as an electron acceptor producing sulfate and ammonia (to be published elsewhere).

A four-gene cluster encodes two multiheme *c*-type cytochromes (TDIS_0609 and TDIS_0606), the iron–sulfur protein similar to B subunits of tetrathionate reductases (TDIS_0608) and the membrane anchor protein (TDIS_0607). The absence of N-terminal signal peptides in these proteins suggests that this oxidoreductase faces the cytoplasmic side of the inner membrane. The specificity of this complex could not be reliably predicted, but location of these genes close to the *nap* operon suggests that activity of this oxidoreductase could be coupled to nitrate reduction.

Two hydrogenases are encoded by the *T. dismutans* genome. An operon of genes TDIS_0913–TDIS_0915 encodes cytoplasmic methyl viologen-reducing hydrogenase MvhDGA. This enzyme, along with cytoplasmic CoB–CoM heterodisulfide reductase encoded by the nearby genes TDIS_0910–TDIS_0912 (*hdrCBA*) forms hydrogen:heterodisulfide oxidoreductase, which catalyzes the reduction of disulfide. In particular, the HdrB subunit (TDIS_0911) contains two cysteine-rich domains with a 4Fe–4S cluster binding motif, involved in the reduction of disulfide bonds (Hamann et al., 2007).

The second hydrogenase, classified as group 1 NiFe enzyme (Vignais and Billoud, 2007), is a membrane-linked respiratory complex that could couple the oxidation of molecular hydrogen to the reduction of quinones and finally the terminal electron acceptor, probably, thiosulfate. The ability of *T. dismutans* to grow with molecular hydrogen as an electron donor and thiosulfate as an electron acceptor was reported in the original description (Slobodkin et al., 2012). The energy could be conserved in the form of a transmembrane proton gradient. This periplasmic complex is encoded close to the hydrogen:heterodisulfide oxidoreductase and includes the small subunit (TDIS_0916) carrying the Tat signal peptide, the large subunit (TDIS_0917) and membrane-bound cytochrome *b* subunit (TDIS_0918) transferring the electrons to the quinone pool.

The genome of *T. dismutans* suggests that formate could be used as an electron donor similar to hydrogen. Formate dehydrogenase of the molybdopterin oxidoreductase family is encoded by the genes TDIS_1816 (catalytic subunit A), TDIS_1818 (iron-sulfur subunit B) and TDIS_1819 (membrane anchor cytochrome *b* subunit C). The presence of an N-terminal Tat signal peptide in FdhA suggests that this formate dehydrogenase is oriented toward the periplasmic site of the internal membrane. However, in the series of additional experiments, we could not demonstrate the ability of *T. dismutans* to grow with formate as an electron donor and elemental sulfur, sulfate, thiosulfate, or nitrate as an electron acceptor.

Another component of the electron transfer chain is NADH-ubiquinone oxidoreductase consisting of the subunits NuoA, B, C, D, H, I, J, K, L, M, and N, encoded by the genes TDIS_1025- TDIS_1014. Genes encoding the subunits NuoEFG were not found in the genome indicating that NADH is likely not an electron donor for this complex. The presence of antiporter subunits suggests that the activity of this complex probably contributes to the generation of a transmembrane proton gradient that could be used by F_1_F_0_ ATP synthase for ATP production.

### Central Metabolic Pathways

The *T. dismutans* genome encodes the complete Embden–Meyerhof pathway of glucose catabolism including glucokinase (TDIS_1571), glucose-6-phosphate isomerase (TDIS_1407), phosphofructokinase (TDIS_0184), fructose 1,6-bisphosphate aldolase (TDIS_0661), triosephosphate isomerase (TDIS_1029), glyceraldehyde-3 phosphate dehydrogenase (TDIS_2022, TDIS_2024, TDIS_2143), phosphoglycerate kinase (TDIS_2021), phosphoglycerate mutase (TDIS_0293), enolase (TDIS_1673) and pyruvate kinase (TDIS_1208). Taking into account that *T. dismutans* is unable to ferment sugars (Slobodkin et al., 2012), the glycolysis pathway probably operates in the reverse direction of gluconeogenesis. Consistently, the enzymes specifically catalyzing the reverse reactions are encoded: phosphoenolpyruvate synthase (TDIS_0764) and fructose-1,6-bisphosphatase (TDIS_0690). The reversible conversion of pyruvate to acetyl-CoA could be performed by pyruvate:ferredoxin oxidoreductase encoded by the genes TDIS_0147- TDIS_0150.

Consistent with the inability of *T. dismutans* to use organic substrates as electron donors, its genomes do not encode the complete tricarboxylic acid cycle, as evidenced by the lack of genes for citrate synthase, succinyl CoA synthetase and succinate dehydrogenase.

*Thermosulfurimonas dismutans* is able to grow autotrophically without organic carbon sources (Slobodkin et al., 2012). Similarly to *D. sulfoexigens*, *T. dismutans* genome encodes a complete Wood–Ljungdahl (the acetyl-CoA reductive) pathway for the fixation of CO_2_, including formate-tetrahydrofolate ligase (TDIS_0997), methylenetetrahydrofolate dehydrogenase/cyclohydrolase (TDIS_0998), methylenetetrahydrofolate reductase (TDIS_1006 and TDIS_0870), methyltetrahydrofolate:corrinoid iron–sulfur protein methyltransferase (TDIS_1009), and the CO dehydrogenase/acetyl CoA synthase complex (Ragsdale and Pierce, 2008). Formate dehydrogenase, the first enzyme of the methyl branch of this pathway is probably encoded by gene TDIS_1010. Contrary to the product of gene TDIS_1816, this formate dehydrogenase lack recognizable N-terminal targeting sequence and is probably located in the cytoplasm. The key enzymes of other known pathways of autotrophic carbon fixation, the reverse tricarboxylic acid cycle and the Calvin–Benson pathways were not identified.

Although the ability of *T. dismutans* to use N_2_ gas as sole nitrogen source for growth was not analyzed at the original description (Slobodkin et al., 2012), its genome contains all genes necessary for nitrogen fixation, including the molybdenum-iron nitrogenase (genes TDIS_0750 and TDIS_0751 coding for subunits α and β, respectively), its regulatory and accessory proteins, all encoded in a single locus (genes TDIS_0746- TDIS_0754).

## Discussion

*Thermosulfurimonas dismutans* S95^T^ is the first known sulfur-disproportionating bacterium of the phylum *Thermodesulfobacteria.* Most representatives of this phylum are sulfate-reducing organisms (Zeikus et al., 1983; Moussard et al., 2004), with the exception of two species of the genus *Caldimicrobium* (Miroshnichenko et al., 2009; Kojima et al., 2016) and *Geothermobacterium ferrireducens* (Kashefi et al., 2002), which reduce thiosulfate and sulfur or Fe(III), respectively, and are incapable of dissimilatory sulfate reduction. *T. dismutans* also does not grow by sulfate respiration (Slobodkin et al., 2012).

**FIGURE 2:**
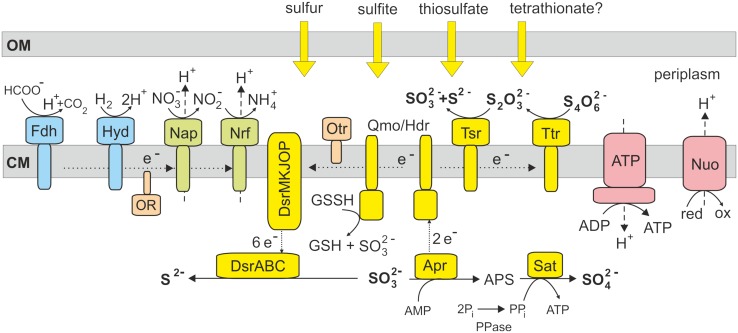
**Model of sulfur metabolism and related pathways in *T. dismutans***. Enzyme abbreviations: Ttr, tetrathionate reductase; Tsr, thiosulfate reductase; Qmo/Hdr, electron transfer complex Qmo and heterodisulfide reductase; Dsr, dissimilatory sulfite reductase; Apr, adenosine-5′-phosphosulfate reductase; Sat, sulfate adenylyltransferase; PPase, pyrophosphatase; Fdh, formate dehydrogenase; Hyd, hydrogenase; Nap, nitrate reductase; Nrf, putative nitrite reductase; OR, oxidoreductase encoded by genes TDIS_0606-TDIS_0609; Otr, octaheme *c*-type cytochrome tetrathionate reductase; ATP, F_1_F_0_ ATP synthase; Nuo, membrane-linked complex comprising subunits NuoA, B, C, D, H, I, J, K, L, M and N of NADH-ubiquinone oxidoreductase. OM, outer membrane; CM, cytoplasmic membrane.

Previous studies of enzymatic activities in deltaproteobacterium *Desulfocapsa sulfoexigens* indicate that sulfite is a key intermediate in the disproportionation of sulfur compounds (Frederiksen and Finster, 2003). The genome of *D. sulfoexigens* contains a full set of genes required for dissimilatory sulfate reduction and the reason why this bacterium does not respire sulfate remains unclear (Finster et al., 2013). Similar to *D. sulfoexigens*, the genome of *T. dismutans* also encodes the complete sulfate reduction pathway that may explain the ability to disproportionate sulfite. The oxidation of sulfite to sulfate could be enabled by reversal of the initial steps of the sulfate reduction pathway, performed by APS reductase and sulfate adenylyltransferase (**Figure 2**). These reverse reactions would result in ATP synthesis and the donation of electrons to the membrane quinone pool. An alternative hypothetical pathway of direct oxidation of sulfite to sulfate by sulfite oxidoreductase (Finster, 2008), found in some sulfur-oxidizing bacteria, seems to be absent in *T. dismutans* as well as in *D. sulfoexigens* (Finster et al., 2013). The reduction of sulfite to sulfide is likely enabled by the dissimilatory sulfite reductase and its accessory proteins, as in the typical sulfate reducers. Thus, *T. dismutans* makes ATP directly by substrate level phosphorylation and also with the aid of ATP synthetase consuming the proton-motive force generated by membrane-linked oxidoreductases.

Disproportionation of thiosulfate likely fits the same pathway with the addition of thiosulfate reductase that splits thiosulfate into sulfite and sulfide (**Figure 2**). The presence of tetrathionate reductase converting tetrathionate into thiosulfate suggests that *T. dismutans* could also grow by the disproportionation of tetrathionate, although this has not yet been studied.

To date, there is no conclusive information on the enzymatic pathways of elemental sulfur disproportionation. It is supposed that sulfite is an intermediate, although the corresponding enzymes(s) performing reactions with elemental sulfur itself were not identified (Frederiksen and Finster, 2003; Finster, 2008). The candidate genes were also not found in the *D. sulfoexigens* genome leading to the suggestion that the oxidation of elemental sulfur to sulfite could depend on the activity of the adenylylsulfate reductase-associated electron transfer complex (Qmo) consisting of subunits A, B, and C, related to the subunits A and E of heterodisulfide reductase (Finster et al., 2013). In sulfate-reducing microorganisms, heterodisulfide reductase catalyzes the reversible reduction of disulfide bonds coupled to the generation of a proton motive force (Mander et al., 2004). Analysis of the *T. dismutans* genome revealed a similar *qmoABC* gene cluster (TDIS_1512- TDIS_1510). It was hypothesized that in the sulfur-oxidizing bacterium *Acidithiobacillus ferrooxidans* heterodisulfide reductase could oxidize disulfide intermediates to sulfite and donate electrons to the quinone pool (Quatrini et al., 2009). Taking into account that the heterodisulfide reductase catalytic site is actually located in HdrB, the involvement of cytoplasmic hydrogen:heterodisulfide oxidoreductase (*hdrCBA*-*mvhDGA*, genes TDIS_0910- TDIS_0915) in this reaction together with membrane-linked Qmo complex could be proposed. As in the case of *Acidithiobacillus* (Rohwerder and Sand, 2003), the actual substrate entering the disproportionation pathway in *T. dismutans* is probably not an elemental sulfur that is poorly soluble and cannot enter the cell, but soluble sulfane-sulfur compound glutathione persulfide (GSSH), which contains a disulfide bond that has been proposed to be cleaved by Qmo/Hdr to produce SO_3_^2-^ and glutathione (GSH). Our observation that *T. dismutans* is able to grow via sulfur disproportionation without direct contact of the cells to solid elemental sulfur further supports this proposal.

Interestingly, *T. dismutans* and *D. sulfoexigens* have several common metabolic pathways besides those related to sulfur metabolism. Both bacteria can grow to grow both autotrophically and diazotrophically, which corresponds to the presence of a reverse acetyl-CoA pathway of CO_2_ fixation and nitrogenase in their genomes. Both genomes suggest a potential for dissimilatory nitrate reduction coupled to elemental sulfur oxidation as an alternative or addition to sulfur-dependent metabolism thus linking sulfur and nitrogen cycles.

Overall, the genome sequence of *T. dismutans* provides new information about the metabolic pathways in this chemolithoautotrophic microorganism. *T. dismutans* was isolated from a chimney of a deep-sea hydrothermal vent where elemental sulfur is an abundant compound and thus bacterial sulfur disproportionation could represent an important process of primary production in such ecosystems. Genomic insights into energy and carbon metabolism of *T. dismutans* will stimulate and facilitate further biochemical and genetic studies required for the understanding of enzymatic pathways of microbial sulfur disproportionation.

## Author Contributions

AM and NR designed the research project and wrote the paper; VK and AS performed the research; AB, AM, and NR analyzed the data.

## Conflict of Interest Statement

The authors declare that the research was conducted in the absence of any commercial or financial relationships that could be construed as a potential conflict of interest.

## Footnotes

1 http://www.cbs.dtu.dk/services/SignalP/

2 http://www.compgen.org/tools/PRED-TAT/

3 http://www.cbs.dtu.dk/services/TMHMM/
